# Perceived predictors of quality of life in patients with end-stage renal disease on dialysis

**DOI:** 10.4102/curationis.v44i1.2251

**Published:** 2021-09-20

**Authors:** Pretty N. Mbeje, Ntombifikile Mtshali

**Affiliations:** 1 Department of Nursing, Faculty of Health Sciences, University of KwaZulu-Natal, Durban, South Africa

**Keywords:** predictors, quality of life, end-stage renal disease, dialysis, renal services

## Abstract

**Background:**

Reduced quality of life (QOL) is associated with shorter survival, and is more marked in patients with end-stage renal disease (ESRD). Predictors of health, which include policymaking, social factors, health services, individual behaviour, biology and genetics, have an impact on the QOL of patients with ESRD. Patients with ESRD in South Africa are cared for in public and private hospitals, with public health institutions characterised by several challenges.

**Objective:**

To explore and analyse the perceived predictors of QOL in patients with ESRD in the three district hospitals that provide renal services in KwaZulu-Natal.

**Methods:**

An exploratory, descriptive qualitative research approach based on grounded theory research design was used and three focus group discussions (FGDs) were conducted. The researcher recruited 18 participants, 6 in each focus group. Three phases of data analysis were followed: open coding, axial coding, and selective coding.

**Results:**

Predictors of QOL that emerged were the geographic location of the patients, accessibility of haemodialysis centres, patients’ adaptation and acceptance of the condition, self-management, support by family members and caregivers and availability of adequately trained nurses.

**Conclusion:**

Predictors of QOL in patients with ESRD are associated with an increased risk of morbidity and mortality resulting from dialysis. The broad range of dimensions of life is negatively affected and requires intervention by the renal team and policymakers to improve the QOL of patients.

## Introduction

A growing body of literature indicates that end-stage renal disease (ESRD) is an increasing global health challenge. It requires advanced intervention by healthcare providers rendering lifelong renal replacement therapy (RRT) in the form of haemodialysis or peritoneal dialysis (Bayoumi & Alwakeel 2012; Gilardino et al. 2018; Lim et al. 2016). End-stage renal disease is particularly pronounced in African countries where there is an estimated overall prevalence of 8% – 16%, which corresponds to nearly 500 million affected individuals of whom 78% (387.5 million) are in low-income to middle-income countries (George et al. 2017). In 2010, an estimated population of more than 2 million worldwide was treated for ESRD, most of whom die because they are declined or cannot access RRT in the form of dialysis (Robinson et al. 2016). The escalating prevalence of ESRD makes it difficult for developing countries to cope with the double burden of communicable and non-communicable diseases (NCD) (Etheredge & Fabian 2017; Isla et al. 2014). The quality of life (QOL) of patients with ESRD is compromised, resulting in shorter survival. The increased morbidity and mortality is an enormous constraint on the provision of healthcare services, especially in developing countries (Naicker 2003). It severely compromises health systems in their obligation, as defined by the World Health Organization (WHO), to ensure the availability of resources in preventing complications and reducing mortality for people with NCD (Puoane et al. 2017).

Predictors or determinants of health are biological, social, economic and environmental factors that vary across individuals’ lifespan and ability to survive in their given circumstances (Hall 2018). There are physical and social determinants of health, but this study focuses on the predictors which have a particular impact on outcomes for QOL. The broad categories of determinants of health include: policymaking, social factors, health services, individual behaviour, biology and genetics (Omotoso & Koch 2018).

Quality of life, as defined by Megari (2013), is the perception that individuals have ‘of their position in life in the context of culture and value systems in which they live and concerning their goals, expectations, standards and concerns.’ Ferrell and Dow (as cited by Lavdaniti & Tsitsis [2014a, 2014b]) describe QOL as encompassing physical, psychological, social and spiritual well-being, whilst Ventegodt et al. (2003) simply define it as ‘good life’. However, even though there is no universal consensus on a definition of QOL, each individual will perceive QOL for themselves so far as they can perform the activities of daily living that reflect physical, psychosocial and spiritual well-being while remaining in control of their disease. Patients with ESRD have poor QOL outcomes as compared to the general population.

Universal health coverage implies equity of access and financial risk protection in the utilisation of healthcare services (Benatar et al. 2018; Fusheini & Eyles 2016). District health systems, mainly consisting of primary healthcare and referral hospitals, are the backbone of health service delivery in low- and middle-income countries, and efficient district linkage in service availability is required at all levels of healthcare delivery (Fusheini & Eyles 2016). Furthermore, it is reported that in sub-Saharan Africa, three-quarters of adult patients die after being put on dialysis as a result of late presentation to healthcare facilities, poor quality of dialysis and cessation of dialysis because of prohibitive costs (Etheredge & Fabian 2017). Whilst unfavourable home conditions in South Africa have been cited as hindering access to care, there has been comparatively little documentation of the challenges encountered by patients with ESRD on dialysis (Etheredge & Fabian 2017; Madala et al. 2014). Assessing and treating symptoms improve patient outcomes; however, QOL should be measured frequently and appropriate interventions should be applied, which seldom happen (Chen et al. 2016). Studies performed in this respect have chiefly been quantitative investigations and retrospective chart reviews (Saad et al. 2015). With this in mind, the present study set out to explore the predictors of the QOL in patients with ESRD in the three district hospitals of KwaZulu-Natal.

## Methodology

### Research design

The qualitative grounded theory approach and exploratory, descriptive design by Strauss and Corbin (1990) were adopted in this study. The inductive nature of the grounded theory approach allowed for the emergence of the new theory from data (Charmaz & Belgrave 2014) as predictors of the QOL for patients with end-stage renal phase was perceived as a relatively unfamiliar phenomenon, especially in the local context of KwaZulu-Natal.

### Setting description

In grounded theory, data collection is conducted in a natural setting as it is central to the generated meaning (Corbin & Strauss 2014; Lincoln & Guba 1985). In this study, the research setting enabled the researcher to obtain in-depth data, strengthening the emerging theory. The researcher selected three public hospitals which comprised of two hospitals at eThekwini district and one at uMgungundlovu district. All three district hospitals offer RRT to patients with ESRD in KwaZulu-Natal. Data collected at these research sites was grounded in diverse settings, as stipulated by Creswell (2014).

### Sampling procedure and participants’ description

In keeping with the grounded theory approach, non-probabilistic purposive and theoretical sampling was applied to ensure that the participants selected would have rich information that would contribute to the study (Corbin & Strauss 2014). This type of sampling requires the researcher to select participants who know about the phenomenon under study. In-patients who were admitted to the renal ward were used for focus group interviews (Chun Tie et al. 2019). A maximal variation sampling strategy was used in which the participants were purposefully chosen to vary in terms of age, gender and race, to provide a complex picture of the phenomenon. Purposive selection of the initial group was followed by theoretical sampling. Expansion of existing codes and emergence of new codes continued until data saturation was reached. The researcher recruited 18 participants, 6 in each focus group, referred to as P1 – P18 in the results section. The participants provided in-depth information on the predictors of QOL in patients with ESRD on dialysis. The quality of data obtained had the potential to be high as the participants were knowledgeable about the study.

### Data collection

In this study, the data collection process and the initial phase of data analysis continued for 7 months (October 2017 – April 2018) using multiple discussion, observations and document analysis methods. Whilst in the haemodialysis unit (HDU), patients were observed as they walked into the unit during dialysis and post dialysis. The observations, document analysis and the Focus Discussion Groups (FDGs) enabled the researcher in the initial phase of data collection to restructure the question guide for other subsequent FDGs. The FDG guide and unstructured probing questions were used to probe the participants to obtain in-depth information on the predictors of QOL in patients with ESRD, thereby increasing the density of the data collected. The FDG guide was used to ensure that all the study’s objectives were met in each data collection session. Each FDG took about 50–60 min, and the participants were observed for any form of discomfort. The discussions were all audio-taped and transcribed to represent the best living conversations. Simultaneously, generous field notes and memos were taken during data collection to provide thick descriptions of the phenomenon under study (Charmaz & Belgrave 2019). Recording all data and keeping them safe for later use, assisted the researcher in writing the memos as the research process unfolded (Corbin, Strauss & Strauss 2014).

### Data analysis

Grounded theory analysis involves the constant comparative method of data analysis (Chun Tie et al. 2019). This process involved constantly comparing emerging data, where codes were compared to codes, codes were compared to categories, categories were compared to categories and meanings were continually reassessed to understand what is going on from the participants’ perspective (Corbin et al. 2014). Maintaining neutrality was very important as the researcher, as an experienced nephrology nurse, was an expert in the renal field; subjectivity did not occur during data collection and the coding process. The three phases of data analysis were followed as outlined by Strauss and Corbin (1990): open coding, axial coding, and selective coding.

Theoretical sampling enabled the researcher to be immersed in the study without any preconceived ideas. In the first stage, the open coding stage, the researcher identified concepts and their properties from data through line-by-line analysis. In this phase, the researcher broke down, examined, compared, and conceptualised data, and grouped data with the same meaning into categories (Corbin & Strauss 2014; Strauss & Corbin 1990). Throughout the analysis process, the researcher continuously consulted with her supervisor to exclude subjectivity and ensure neutrality and authenticity of the phenomenon under study. In the second stage of data analysis, the axial coding stage, the researcher described all the concepts relating to predictors of QOL in patients with ESRD in terms of properties and dimensions. The coding paradigm was used to connect categories found during open coding (Wiesche et al. 2017). The process of connecting related categories under one category helped reduce the number of initial categories to be meaningfully and hierarchically organised (Corbin et al. 2014). The third stage, which is the selective coding, involved integration, unification and refinement of the emerging theory, verifying and validating categories against data and focused literature (Wiesche et al. 2017). The researcher was able to capture the core categories that emerged, and this was followed by the process of reduction and constant comparison whilst identifying relationships between and amongst the concepts. This process led to the emergence of a storyline as defined by Corbin et al. (2014) on the predictors of the QOL in patients with ESRD.

### Rigour

Following the criteria outlined by Lincoln and Guba (1985), trustworthiness was ensured by credibility, dependability, transferability and confirmability. Conformity with these criteria reinforced the authenticity and quality of the study findings. The emerging findings were double-checked, and a co-coder was used to confirm the emerging codes and categories for credibility. The study’s dependability was ensured by triangulating data sources and validating transcribed interviews; and the research supervisor double-checked the emerging codes and categories. Transferability was ensured by providing thick descriptions of the study context, settings, procedures and findings. Confirmability was ensured through matching collected data against the participants’ original understanding of the phenomenon of interest in the study.

## Results

Data analysis led to the emergence of four categories of perceived determinants of QOL in patients with ESRD: social factors, personal factors, geographical location and health system (see Table 1). A growing body of literature has indicated that ESRD is a global health challenge. It continues to rise progressively and requires advanced intervention by the healthcare providers who will render lifelong RRT in the form of haemodialysis or peritoneal dialysis (Bayoumi & Alwakeel 2012; Gilardino et al. 2018; Lim et al. 2016).

**TABLE 1:** Perceived determinants of quality of life categories, sub-categories and dimensions.

Categories	Sub-categories
1. Social factors	Care givers’ support
	Patients’ financial status
	Ability to adapt to change in culturally ascribed social roles
2. Personal factors	Physical health status
	Healthy coping mechanisms
	Existence of comorbid conditions
	Level of health literacy
	Age and body image
3. Geographical location	Distance from the haemodialysis unit
	Quality of sleep
4. Health systems related factors	Infrastructure in terms of quality and quantity
	Prioritisation of patients from remote areas
	Government’s commitment to patients’ access
	Human rights compromised
	Supply-side driven approach versus demand-driven approach

### Social factors

The sub-categories that emerged under social factors that determine the QOL in patients with ESRD were caregivers’ support, patients’ financial status, and ability to adapt to change in culturally ascribed social roles.

#### Caregiver’s support

Data showed that the availability of caregivers influenced the QOL of patients with ESRD.

Participants with self-care deficiency reported that the caregivers’ support from their family members played an essential role in their survival. Responses confirmed that most participants had good social support, which positively impacted their survival and RRT compliance. Another reported form of support was transport to the hospital for dialysis or appointments. Emotional and financial support and provision of transport are reflected in the following participant comments:

‘my family has been supportive through my journey, and this means a lot to me coping with this condition …’ (P11, female patient, Hospital B, FDG 3)

‘I fully depend on my mother for assistance with everything I need.’ (P3, female patient, Hospital A, FDG 1)

‘… the support that I get from my family makes me feel normal like every other person irrespective of illness …’ (P15, male patient, Hospital C, FDG 3)

‘I can call my neighbours anytime if I cannot get transport to go for dialysis or doctor’s appointment, and they always help if available …’ (P7, male patient, Hospital B, FDG 2)

#### Patients’ financial status

The data sources showed that patients’ financial status affected QOL in patients with ESRD. Participants revealed that most of them were unemployed because of their dialysis schedule (mostly three times a week) and frequent hospitalisation. Even temporary employment was unsustainable because of the side effects of dialysis such as easily getting tired. Participants were financially dependent on their families as a result of unemployment. These financial issues are illustrated in the following comments by the respondent:

‘I lost my job as a result of frequent hospitalisation and going for dialysis three times a week …’ (P1, male patient, Hospital A, FDG 1)

‘… even if I get a temporary job, I easily get tired and cannot keep it …’ (P12, male patient, Hospital B, FDG 2)

‘Surviving by my grandmother’s pension all the time …’ (P6, female patient, Hospital A, FDG 1)

#### Ability to adapt to change in culturally ascribed social roles

Participants reported lowered self-esteem from alteration in their culturally ascribed role when unable to assume their normal social position. Some participants reported that unemployment resulting from scheduled haemodialysis was a significant cause of change in their social role, especially if they were at an age when they would expect to be independent. Other forms of role change dependency reported were children taking care of parents at an early age, and men providing care to sick women. In most households, the only source of income was an old-age pension, causing drastic change in social roles as culturally ascribed. These points are reflected in the following respondent comments:

‘Surviving by my grandmother’s pension all the times when I am at the age of being employed …’ (P6, female patient, Hospital A, FDG 1)

‘… my kids have to ensure that the house is clean and they have cooked before going to school …’ (P17, female patient, Hospital C, FDG 3)

‘… it was not an easy task when my husband had to bathe me and do the bag change when I could not …’ (P3, female patient, Hospital A, FDG 1)

‘I am old enough to have my own family, but my mother is taking care of me, ensuring that I have something to eat and money to go to the hospital …’ (P3, female patient, Hospital A, FDG 1)

‘My wife had to look for a job, and now I am taking the role of cleaning and cooking since our kids are still young …’ (P14, male patient, Hospital C, FDG 3)

### Personal factors

Personal factors that emerged as predictors of QOL in patients with ESRD are detailed below.

#### Physical health status

Being in a good physical state of health aside from renal illness contributed positively to QOL. The participants reported poor physical health, most often on peritoneal dialysis, since they faced the challenge of recurrent infections such as peritonitis, tract abscess and even blocked dialysis catheter. These problems result in patients being hospitalised and sometimes surgical intervention, with negative consequences for their physical condition. Change in compliance with renal diet affected most of the respondents, with drastic weight loss in some instances. However, good physical health aside from the renal illness indicated improved QOL. The following respondent comments illustrate these points:

‘… with peritoneal dialysis, you experience many problems like peritonitis, tract abscess, which is very painful …’ (P7, male patient, Hospital B, FDG 2)

‘… the main problem with peritoneal dialysis is infection. Sometimes you get the infection even …’ (P11, female patient, Hospital B, FDG 2)

‘… I do not eat well and have lost so much weight …’ (P10, female patient, Hospital B, FDG 2)

#### Healthy coping mechanisms

Healthy coping mechanisms emerged as determinants of good health in relation to coping with the changes and challenges that participants encountered in undergoing dialysis. Adaptability was mainly expressed in relation to the beneficial influence of religious belief, family support and acceptance of illness in one’s life. This results in acceptance of illness which facilitates treatment adherence. The following extracts illustrate these responses:

‘… now I have faith that one day God will help me to recover fully …’ (P3, female patient, Hospital A, FDG 1)

‘If it were not for the support given by my family, I would not be coping through all the difficulties that I face …’ (P16, female patient, Hospital C, FDG 3)

‘… you just have to accept that you have kidney problems and …’ (P10, female patient, Hospital B, FDG 2)

#### Existence of comorbid conditions

The common comorbid conditions in patients with ESRD are hypertension and diabetes mellitus. The presence of comorbid diseases increases the symptom burden in patients with ESRD on dialysis. Most participants had either diabetes or hypertension as comorbid conditions, and some had both. The comorbidity negatively affects QOL as it requires increased self-care in terms of treatment and diet adherence, and lifestyle modification. These issues are illustrated in the following respondent comments:

‘I was doing well with diabetes, and high BP, kidney failure turned my life upside down …’ (P12, male patient, Hospital B, FDG 2)

‘… my blood pressure is always high, but blood sugar is not a problem; what worries me most is the catheter infection …’ (P5, female patient, Hospital A, FDG 1)

‘… I changed my diet and cannot go to the gym anymore …’ (P6, female patient, Hospital A, FDG 1)

‘… I have to ensure that I change my bag four times a day without fail …’ (P11, female patient, Hospital B, FDG 2)

#### Level of health literacy

The level of health literacy emerged as a factor affecting QOL in patients with ESRD. Patients on dialysis are given many booklets and other information that they are expected to understand for compliance and adaptation in the treatment regime. Study participants noted that they were taught about the importance of environmental hygiene when undergoing peritoneal dialysis and about fluid and dietary restrictions and symptoms management and their implications. The level of health literacy significantly influences the likelihood of adherence to the advice given by healthcare providers, especially concerning dietary and fluid restriction. The following respondent comments are illustrative:

‘Hygiene is paramount when you are on dialysis … everything is to be clean, washing hands thoroughly and the surface where you do your bag change has to be spotless clean …’ (P10, female patient, Hospital B, FDG 2)

‘Complying with fluid and diet intake as taught by nurses is crucial …’ (P12, male patient, Hospital B, FDG 2)

‘… It is important to know the problem you may encounter and what to do when at home …’ (P9, male patient, Hospital B, FDG 2)

‘… Knowledge of your illness makes you understand the importance of dialysis no matter …’ (P5, female patient, Hospital A, FDG 1)

#### Age and image

Age and image emerged as factors that affected self-esteem and self-actualisation chiefly of the younger patients on dialysis. Most participants, but especially the younger ones, revealed that they were stressed by changes in body image as a result of dialysis side effects leading to the darkened complexion, loss of weight and increased frailty. The bone weakness affects gait, and some patients may need to use crutches, walking sticks and even wheelchairs, depending on the severity of the bone disease. Younger participants were embarrassed about the change in their body image, especially if questioned about it. Conversely, older patients were less concerned about image issues and more likely to accept these consequences. The following comments illustrate these personal image concerns:

‘I have become so dark in a way that others still cannot recognise me …’ (P3, female patient, Hospital A, FDG 1)

‘… the dialysis catheter that is hanging on my abdomen embarrasses me when I undress in front of others as I have to explain when asked …’ (P9, male patient, Hospital B, FDG 2)

‘I have lost so much weight and look completely different …’ (P4, male patient, Hospital A, FDG 1)

‘I am so young, but I cannot walk properly as I have to use the crutches since all my joints are weak … wanted to be a soccer star, but my dreams were shattered by dialysis …’ (P2, female patient, Hospital A, FDG 1)

### Geographical location

Distance from the HDU affects QOL in patients with ESRD. Patients in urban areas closer to HDUs are at an advantage over those who travel from remote areas. The following comments made by two of the respondent illustrate characteristic problems for patients faced with long-distance travel: fatigue and swollen limbs, and time away from their families:

‘… travelling long-distance makes me feel so tired, and my legs are always swollen …’ (P13, female patient, Hospital C, FDG 3)

‘… I hardly spend time with my family because I am always on the road travelling to and from haemodialysis …’ (P15, male patient, Hospital C, FDG 3)

#### Quality of sleep

Regular sleeping patterns were reportedly affected as result of the transport arrangements needed by the patients for visiting their HDU – in some cases requiring the patient to stay overnight in the neighbouring hospital. With thrice-weekly dialysis, these arrangements caused severe sleep disruption and heightened exhaustion for the affected patients when restful sleep is critical for healing and recovery. The problem is apparent in the following participants comments:

‘If you have to come to the hospital three times a week, you have to sleep in the hospital three times a week …’ (P6, female patient, Hospital A, FDG 1)

‘… we sleep in the local hospital benches while waiting for the bus and sleep on the way to dialysis hospital, and arrived back home late at night three times a week …’ (P18, female patient, Hospital C, FDG 3)

### Health system-related determinants of quality of life

#### Infrastructure quality and quantity

Study responses indicated that infrastructure for the provision of quality care was a significant determinant of QOL in patients with ESRD. Personnel resources, including staff with renal expertise and dialysis equipment resources, are both crucial factors for rendering quality care to patients on dialysis.

The renal psychologist’s role is to support the patient at all stages of treatment to prevent psychological complications that may affect the patient’s treatment and QOL. Participants’ responses confirmed that patients identified with dietary problems were referred to the renal dietician for comprehensive professional advice on dietary requirements and adherence, and those patients requiring special attention were referred to the relevant healthcare professionals. Nurses provided health education to ensure that patients had the necessary knowledge and skills for adherence, self-care and self-management of dialysis side effects. Similarly, responses underlined the critical role played by other members of the multidisciplinary renal team. The comments below reflect participants’ experiences of the treatment infrastructure:

‘… we are seen by the renal doctors almost once a month …’ (P17, female patient, Hospital C, FDG 3)

‘… the nurses check our blood results and change our dialysers if there is a need …’ (P17, female patient, Hospital C, FDG 3)

‘I cannot go anywhere if I am sick; I must only come to Greys because they have nurses and doctors that understand my illness …’ (P14, male patient, Hospital C, FDG 3)

‘I was advised by nurses on which bags to use for dialysis when drainage is poor … the social worker is in the process of making an arrangement for me to get a grant.’ (P14, male patient, Hospital C, FDG 3)

Restricted availability of haemodialysis machines and insufficient renal experts emerged as negatively affecting QOL in patients with ESRD. Participants noted that there were prescribed times for dialysis sessions which meant that patients could only come for treatment when a dialysis machine was available. This compromised QOL, particularly for those in remote areas where there were no renal experts to deal with renal challenges that a patient might be faced with. The following respondent comments indicate some of these problems:

‘There is no extra machine, and you cannot just come in anytime …’ (P5, female patient, Hospital A, FDG 1)

‘… even if you are short of breath and need dialysis urgently, there is no standby machine. Instead, you wait for one patient to finish dialysis …’ (P13, female patient, Hospital C, FDG 3)

‘… if you are sick, no matter where you are, you must come to your dialysis hospital to be seen only by renal doctors and renal nurses …’ (P9, male patient, Hospital B, FDG 2)

#### Prioritisation of patients from remote areas over local patients

It emerged that dialysis unit patients arriving by ambulance from more remote hospitals were given priority. This resulted in local patients spending the whole day, sometimes even the night, in the dialysis unit, depending upon the number of patients arriving by ambulances. This compromised their QOL as the unpredictable schedule for dialysis made it impossible for them to plan their day. The problem is made apparent in the following comments:

‘… the patients who arrive at the haemodialysis unit from far are prioritised over us as local patients …’ (P9, male patient, Hospital B, FDG 2)

‘… as local patients, we now end up spending the whole day in the HD unit and sometimes dialyse at night and leave the unit in the morning …’ (P10, female patient, Hospital B, FDG 2)

‘I cannot plan anything for dialysis day even if it is urgent because of the unknown time of dialysis …’ (P14, male patient, Hospital C, FDG 3)

#### Government commitment to access for patients

Government commitment to the accessibility of treatment emerged as a positive determinant of QOL in patients with ESRD. One access feature reported was that patients from remote geographical areas spending the night in their local hospitals to avoid missing the government buses that transport them to dialysis units. The patients were also given tea and lunch whilst on dialysis so that they were not affected by lack of nourishment during the long hours they spent travelling to dialysis units:

‘If you have to come to the hospital three times a week, you have to sleep in the hospital three times a week …’ (P6, female patient, Hospital A, FDG 1)

‘… we go to our local hospitals to sleepover so that we do not miss the buses to dialysis units …’ (P17, female patient, Hospital C, FDG 3)

## Discussion

Care, love and support from family members emerged as the important social factors for patients with ESRD in the study. This may take the form of physical support when patients cannot perform their daily living activities and have to depend on others for assistance. In this study context, patients were consistently in need of support as their illness was unpredictable and involved frequent hospitalisation and exposure to dialysis-related surgical interventions. This confirms that psychosocial support from caregivers for patients on dialysis positively affects their QOL (Oyegbile & Brysiewicz 2017). Additionally, it is in line with Maslow’s hierarchy of basic human needs, which are: physiological, safety and security, love and belonging, self-esteem and self-actualisation (Fallatah & Syed 2018). Figure I illustrates the social support as a predictor of health, in the form of complete engagement of family and loved ones in helping to meet the patient’s needs, falling within Maslow’s concept of ‘love and belonging’.

A further social factor that emerged as predicting QOL in patients with ESRD was the patient’s financial status. Hospitalisation and dialysis schedules for patients on dialysis prevented them from maintaining their jobs, resulting in financial dependence on their families with negative consequences for QOL. This finding corresponds with the findings by Gerasimoula et al. (2015), who reported that patients on dialysis were affected by unemployment because they were unable to keep up with their jobs because of excessive time demand put up by regular dialysis sessions they had to undergo (primarily three times a week), resulting in financial constraints that may cause failure to adherence to dialysis schedules. Similarly, Anees et al. (2014) found that financial status affected the QOL of patients with ESRD and all other physical, social and psychological aspects of their lives. Unemployment has a direct physical effect on patients when they cannot provide themselves with a proper diet and are unable to keep up with the scheduled appointments.

The ability to adapt to changes in the culturally ascribed social roles emerged in various ways as a social factor that affected QOL in patients with ESRD. QOL in male patients who had been breadwinners were affected by the role change when they became dependent on their wives or an elderly pension recipient in the family. A different example of this role change with problematic psychological consequences for QOL was when the male partners were put in the position of intimate caregivers for the female patients. Most of the study participants agreed that social support played an essential role in their survival and enabled them to cope with the RRT that they were receiving. This corroborates the conclusion arrived at by Clark et al. (2014) in their study that there is an association between low social support and non-adherence. It likewise confirms with the findings of Varghese (2018) that social support from the family and friends profoundly impact the QOL in patients with ESRD, which increases compliance and lifestyle adjustment.

**FIGURE 1:**
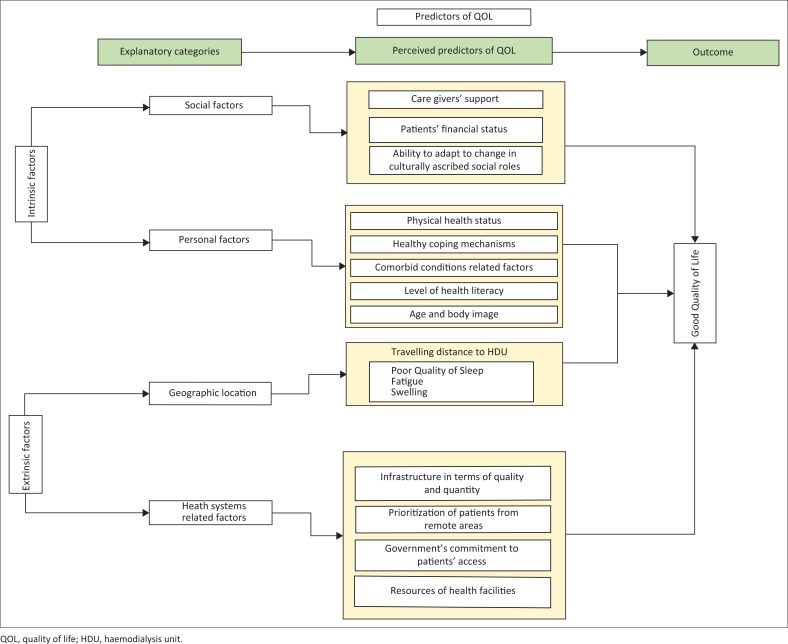
Predictors of quality of life.

Under the personal factors that emerged as predictors of QOL (see Figure 1), good physical health aside from the renal illness indicated good QOL. Conversely, poor physical health indicated poor QOL, in that the patients may be unable to meet the needs of daily living. Likely health issues are diabetes and hypertension, which are major risk factors affecting QOL in sub-Saharan Africa and critical contributing factors to chronic kidney disease (Kilonzo et al. 2017). Madala et al. (2014) cite that hypertension, diabetes and glomerulonephritis are the major causes of chronic kidney disease and ESRD in South Africa. The escalating prevalence of ESRD makes it difficult for developing countries to cope with the double burden of communicable and non-communicable diseases (Isla et al. 2014); in this study, participants had diabetes mellitus or hypertension or both, with a significantly detrimental impact on their QOL.

Healthy coping mechanisms are essential for patients with ESRD in their adaptation to dialysis and its challenges. In this study, coping or adaptation, which is the central concept used in the Roy Adaptation Model – was apparent in accepting illness accompanied by strong religious beliefs. Spirituality plays a vital role in alleviating suffering and promoting well-being, especially in patients with chronic illnesses; spiritual care should, therefore, be provided by the healthcare professionals (Chan et al. 2014; Megari 2013) Similarly, Kharame et al. (2014), in their study on spiritual well-being for QOL in Iranian haemodialysis patients, concluded that spirituality is an autonomous predictor of QOL, which is the intrinsic force that enables patients to survive and cope with all the challenges that they encounter; spiritual well-being was found to be a good predictor of a better QOL. They further recommended that religious organisations should take on the responsibility of providing support for patients with chronic diseases.

The existence of comorbid conditions was identified as a predictor of poor QOL in patients with ESRD on dialysis. All diseases affect the body system, which is why adherence to treatment and dietary requirements is necessary. The study findings revealed that most participants had underlying conditions which were leading causes of ESRD. Patients had diabetes or hypertension or both, or other conditions that add to the burden of illness for patients with ESRD. Multi-morbidity worsens QOL, thereby increasing the mortality rate. This puts a considerable constraint on health systems, especially in developing countries, to the detriment of healthcare services provision for the population in need (Naicker 2003). Stabilisation of the uncontrolled comorbid conditions, treatment of recurrent infections and surgical interventions are associated with frequent hospitalisation and clinical outcomes, seriously affecting the emotional well-being of patients and their families (Lim et al. 2016).

The level of health literacy, which emerged in this study as another personal factor, relates to Orem’s concept of self-management, which emphasises the importance of self-care for improvement of well-being and maintenance of the health of an individual (Audulv et al. 2016). Patients with ESRD on dialysis are given a lot of information by the renal team that will enable them to survive and cope with renal failure. However, this rich and extensive information requires patients to clearly understand the disease and its management, including when and how to solve problems that may be encountered. The most crucial emphasis for dialysis patients is diet and fluid restriction. Removal and control of excess fluid from the body calls for engagement and self-discipline on the part of the patient, which is the cornerstone of fluid management in patients with ESRD (Chironda & Bhengu 2016). Diet and fluid restrictions are a considerable challenge for patients with ESRD, and it takes a long time for them to adapt to their illness, causing deterioration in their QOL. Reinforcement of dietary and fluid restrictions is fundamental to improving clinical outcomes and chances of survival, but no matter how well the patients are informed, adherence is difficult, especially in hot weather when they become very thirsty (Chironda & Bhengu 2016). Through self-care, individuals can take care of themselves, maintaining their lifestyle for optimum health, but when a stage is reached where self-care is not sufficient, nursing/professional intervention becomes a necessity (Ali 2018).

Age and body image emerged as predictors of QOL in patients with ESRD on dialysis. Psychiatrist Paul Schilder, in 1935, described body image as an individual’s overall sense of their physical appearance (McDermott et al. 2015). This study revealed that younger patients, especially females, had higher levels of dissatisfaction with body image, which affected their QOL. Younger patients, in particular, were affected by the scars from frequent surgery that were visible when they were getting dressed; this diminished self-esteem, with a detrimental effect on QOL. Similarly, feelings of shame, low self-esteem and stress caused by negative perceptions of body image may lead to eating disorders with further detriment to QOL (McDermott et al. 2015). Bone mineral density in dialysis-dependent patients is also significantly lower than for the general population, and in younger patients, this frailty could be a serious hindrance to pursuing their personal aspirations (Nakanishi et al. 2018). Conditions that dialysis patients may suffer from are: osteoarthritis, fractures and osteonecrosis, which may in some cases result in total knee replacement surgery (Nakanishi et al. 2018; Ottesen et al. 2018).

Distance from HDUs, as indicated in Figure 1, meant that some patients had to leave home early in the morning or the previous night to avoid missing the transport to dialysis institutions. Long travelling time whilst remaining seated also meant that patients arrived for treatment, exhausted and with swollen limbs. Thrice-weekly dialysis sessions, spending up to 4 h on the haemodialysis machine plus travelling time, all lead to patients spending a considerable time away from their families, and this affects their QOL. In this study, the resources for dialysis were available only in two selected districts utilised by the whole province. Travelling time to access health services for haemodialysis patients in the study compromised their QOL physically, psychologically, socially and most especially economically.

Patients from remote geographical areas experience poor quality of sleep in addition to sleep disturbance caused by multi-morbidity. Study findings revealed that patients slept over in their local hospitals – leaving home the night before and sleeping on hospital benches whilst waiting for the escort buses. They would sleep again in the bus whilst travelling to the dialysis hospitals and again during dialysis. This sleep disturbance contributes to swelling, fatigue and exhaustion, which also delays recovery. Poor sleep quality affects physical and psychological well-being (Milrad et al. 2017; Saad et al. 2015); sleep deprivation is linked with worsening of diseases and inflammatory processes resulting from the impairment of the immune system (Milrad et al. 2017).

Health-system-related predictors of QOL in patients with ESRD on dialysis that emerged in the study were quality and quantity of infrastructure, prioritisation of patients from remote geographical areas and government commitment to ensuring patients’ access to health institutions. Availability of health services of the renal team is a fundamental requisite for QOL in patients on dialysis. The renal nurses, nephrologists, psychologists, dieticians and social workers who form the renal team provide holistic care to patients with ESRD. Seeing the nephrologist, even if only once a month, as was reported in this study, positively affects patients’ QOL. Nurses, who also initiate and discontinue dialysis, play an essential role in providing patients with information and health education that helps to ensure adherence to treatment and dietary requirements and equips them with the knowledge and skills they need to take care of themselves. Patients in need of dietary information for compliance with renal diet are referred to the renal dietician who involve the family members in acquiring the skills for proper renal diet preparation (Chironda & Bhengu 2016). This effective reciprocal interaction between the renal team members positively impacts the QOL in patients with ESRD (Imamura et al. 2019).

Integrated service delivery strengthens collaboration between service agencies in jointly providing support, services and interventions to improve the health outcome of all their clients. Collaboration is strengthened by adequate and appropriate responses, resolving duplication that may occur, developing a single plan for the client, building understanding and knowledge sharing (Zhuwau et al. 2017). Support from renal experts is vital to ensure efficient management of patients with ESRD that will improve their QOL. This study reinforces the point that Africa as a whole faces a challenge of insufficient healthcare professionals with expertise in renal care, coupled with the unavailability of haemodialysis machines in most health institutions (Isla et al. 2014).

Moreover, the issue that emerged in this study was that patients from outside the catchment area of the HDU were given priority over the local patients (see Figure 1). Patients who went through dialysis at night were kept in the unit overnight and left the unit in the morning to go back home through local transport. The patients subject to this arrangement were deprived of quality time with their families. The province of KwaZulu-Natal has a total population of around 11 million, in which there are approximately 2,000 patients with ESRD who are on RRT (Davids et al. 2017). Resources for patients with ESRD are mainly in the eThekwini and uMgungundlovu districts. Transport and geographical issues affect both rural and urban patients. An American study on the transportation of patients with ESRD (Park & Kear 2017) found that transportation limitations had a negative impact on their QOL. Contrary to this present study, in which patients were provided with transport free of charge, the American study found that patients were missing dialysis sessions because of poor transportation, which was associated with excessive usage of the critical care and the emergency units.

In South Africa, the government is committed to quality care of patients with ESRD on dialysis and access to health services is not compromised. In this study context, patients going for dialysis were accommodated in their local hospitals do that they did not miss the buses taking them to the dialysis HDUs. Lunch packs were provided, where necessary, to provide sustenance during the travelling time, and patients were also given tea and lunch during the dialysis sessions. These interventions ensured that patients did not miss their dialysis schedules and doctors’ appointments. Access to health services becomes a challenge when patients are primarily from remote or rural areas with limited or non-existent access to health services (Zhuwau et al. 2017). As this is made clear in Maslow’s hierarchy of needs, access to health services is a basic need for human beings, and this study exemplified the availability of dialysis units in health institutions.

## Limitations

The large population of patients with ESRD in KwaZulu-Natal are undergoing treatment in the private hospitals, whereas this study focussed on two district hospitals that provide renal services in KwaZulu-Natal.

## Recommendations

The study recommends using press, media and community gatherings to raise public awareness about renal failure so that society is more knowledgeable about renal failure and its complications. Education of healthcare providers at all levels of care will assist in early detection of renal failure, and early referral and management will help prevent patients from reaching the stage of ESRD where dialysis is required. Having the knowledgeable multidisciplinary team on board will provide holistic care to patients, promote recovery and adaptation to a new lifestyle and promote effective healthcare services delivery.

## Conclusion

The findings of the current study indicate that the determinants of health affect QOL in patients with ESRD in the district hospitals of KwaZulu-Natal. Policymakers may utilise the findings generated from this study in formulating strategies to improve QOL in patients with ESRD. Active involvement of the renal multidisciplinary team members, healthcare workers in the primary health care (PHC) settings and partnership with the private sector will have a crucial impact on the improvement of QOL, thereby reducing the adverse effects of dialysis.
